# DECAF Score in Predicting Outcomes of Acute Exacerbation of Chronic Obstructive Pulmonary Disease: An Observational Study

**DOI:** 10.31729/jnma.8903

**Published:** 2025-03-31

**Authors:** Amit Prajapati, Yubaraj Sharma, Suman Thapa, Sadina Devkota

**Affiliations:** 1 Department of Internal Medicine, Bhaktapur Hospital, Dudhpati, Bhaktapur, Nepal; 2 Department of Internal Medicine, Patan Academy of Health Sciences, Lagankhel, Lalitpur, Nepal; 3 Department of Internal Medicine, Dhading Hospital, Dhading, Nepal

**Keywords:** *acute exacerbation*, *COPD*, *DECAF score*

## Abstract

**Introduction:**

Acute exacerbation of chronic obstructive pulmonary disease (AECOPD) can often lead to hospital admission and has the potential to be fatal. Lack of prognostic research in exacerbation requiring hospitalization that can accurately predict inhospital mortality is a challenge. This study aims to assess value of the DECAF (Dyspnea, Eosinopenia, Consolidation, Acidemia and Atrial fibrillation) score as a clinical prediction tool for inhospital mortality, need of intensive care unit (ICU) stay and risk stratification in patients with Acute exacerbation of chronic obstructive pulmonary disease.

**Methods:**

This is an observational cross-section, hospital based study conducted from April 2022 to February 2023 at a tertiary care centre. The patients admitted with the diagnosis of acute exacerbation of chronic obstructive pulmonary disease were included in the study and their DECAF score were calculated. Patients were followed up during hospital stay and their outcome were recorded. The prognostic value of DECAF score was assessed by area under receiver operator characterstics curve.

**Results:**

There were 83 patients enrolled in the study out of which 13 (15.66%) died in the hospital and 20 (24.09%) required ICU stay. The area under receiver operator characteristic curve value for mortality owas 0.89 and that for intensive care unit stay was 0.84.

**Conclusions:**

This study shows that DECAF score is a good predictor of inhospital mortality and ICU admission.

## INTRODUCTION

Acute exacerbation of chronic obstructive pulmonary disease (AECOPD) significantly impacts global healthcare, comprising one in eight hospital admissions and is linked to declining lung function and heightened mortality risk.^1^ Inhospital mortality rates for AECOPD range from 4.4% to 25%, with survivors facing high one-year mortality risks.^2^ In Nepal, Chronic Obstructive Pulmonary Disease (COPD) patients experience frequent exacerbations and hospitalizations,^3^ with inhospital mortality rates varying from 6% to 20%, underscoring the local healthcare burden.^4,5^

Despite established prognostic tools like the Body Mass Index, Airflow Obstruction, Dyspnoea, Exercise (BODE) Score for stable COPD, predicting mortality during AECOPD hospitalizations remains underexplored.^6^ The DECAF score, introduced by Steer et al. is easy to use at the bedside and predicts in-hospital mortality using indices that are routinely available upon admission.^7^

This study aims to assess the prognostic value of DECAF score based on inpatient mortality and need of ICU stay in patient with AECOPD with the goal of enhancing treatment outcomes, ensuring appropriate palliative care, and efficient use of resources.

## METHODS

This is an observational cross-section, hospital based study conducted between April 2022 and February 2023 at Patan Academy of Health Sciences which is a tertiary level hospital situated at Lalitpur, Nepal. The ethical approval was taken from institutional review committee (Reference number: PMM2204051607).

Patients who were more than 40 years and admitted with the diagnosis of AECOPD and providing consent were included in the study. Patients with history of bronchial asthma, bronchiectasis, interstitial lung disease, lung cancer, primary diagnosis other than AECOPD, leaving against medical advice, discharged on request, admitted for palliative care or change of diagnosis during the course of ho

Sample size was calculated using web based statistical software with parameters tabulated on easyROC. The sample size calculation was based on statistical analysis by Obuchowski et al.^8^

Data source for AUROC(0.83) was used from previous study.^9^ Allocation ratio was set based on percentage of inpatient mortality from previous study at Western Regional Hospital, Pokhara, Nepal.^5^ The negative outcome of the study was 6% and value of the allocation ratio was calculated by dividing positive outcome by negative outcome i.e. 94/6=15.6. The desired minimum sample size was 83 with at least 5 mortalities. Convenience sampling was done till desired minimum sample size was reached.

Patients were defined as the case of COPD on the basis of their history- GOLD key indicators^10^ (dyspnea, chronic cough, chronic sputum production, history of exposure to risk factors), physical examination and/or radiological findings. An exacerbation of COPD was defined as an acute worsening of respiratory symptoms (including increased dyspnea, increased sputum volume or purulence, cough or wheeze) that results in additional therapy.^10^ Inpatient mortality was defined as mortality due to any cause in a patient admitted with AECOPD during that admission. Need of Intensive Care Unit (ICU) stay was defined as admission or transfer to ICU during that admission period. Institutional protocol of COPD of Patan Hospital, has defined supporting points for considering ICU care. However, admission and transfer to ICU were based on treating clinician discretion. Data was collected based on the structured proforma once patient was admitted to medicine department. All patients were subjected to thorough medical history and full clinical examination. Routine laboratory investigations including complete blood count, eosinophil count, ABG (arterial blood gas) analysis, chest X-ray or CT (computed tomography) findings and presence of atrial fibrillation (AF) in ECG were recorded. Breathlessness was graded according to eMRCD (extended medical research counsel) scale. DECAF score was calculated and the outcome of inhospital death or discharge were noted (Table 1).

The DECAF score is a prognostic tool used to assess the severity and predict outcomes in patients with acute exacerbations of chronic obstructive pulmonary disease (COPD). It consists of five key variables, each assigned a specific score. Dyspnea is categorized based on the extended Medical Research Council Dyspnea (eMRCD) scale, with a score of 1 for eMRCD 5a and 2 for eMRCD 5b. Eosinopenia, defined as an eosinophil count of <0.05 × 10^9^/L, is assigned 1 point. The presence of consolidation on imaging, acidemia with a pH below 7.3, and atrial fibrillation each contribute 1 point. The total DECAF score ranges up to 6, with higher scores indicating increased mortality risk and poorer prognosis in COPD exacerbations.^7^

Data was entered and analyzed in Microsoft Excel spreadsheet and MedCalc Version 20.104 software. Area Under the Receiver Operator Characterstic Curve (AUROC) of DECAF score was calculated and its sensitivity and specificity were used to determine optimal cutoff and further classify into low, moderate and high-risk groups.

## RESULTS

There were 83 patients included in the study, mean age of the population was (69.1±9.5) years and female population comprising of 57 (68.67%). There were 32 (38.55%) patient whose Extended Medical Research Council Dyspnea (eMRCD)Scale was 4 and among 83 patients, 29 (34.93%) has DECAF score of 1 (Table 1).

**Table 1:** Baseline characteristics of the patients. (n=83).

Varaible	n (%)
**Sex**	
Male	26 (31.33)
Female	57 (68.67)
**eMRCD**	
1	1 (1.20)
2	7 (8.43)
3	24 (28.91)
4	32 (38.55)
5a	18 (21.68)
5b	1 (1.20)
**Eosinopenia(<50/mm^3^)**	31 (37.34)
**Radiographic consolidation**	38 (45.78)
**Acidemia**	28 (33.73)
**AF**	4 (4.81)
**DECAF Score**	
0	18 (21.68)
1	29 (34.93)
2	21 (25.30)
3	14 (16.86)
4	1 (1.20)
5	0
6	0
Age, mean years±S.D	69.1±9.5

AF = Atrial Fibrillation; eMRCD = Extended Medical Research Council Dyspnea Scale; S.D = Standard Deviation.

**Figure 1:**
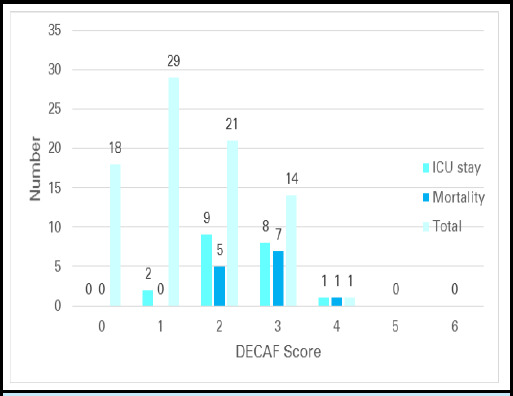
ICU Stay and Mortality in patient of AECOPD based on DECAF Score (n=83).

There were 13 (15.66%) mortality during hospital stay, median DECAF score of those were 3. Similarly, 20 (24.09%) patients required ICU stay and their median DECAF score was 2 (Figure 1).

**Figure 2:**
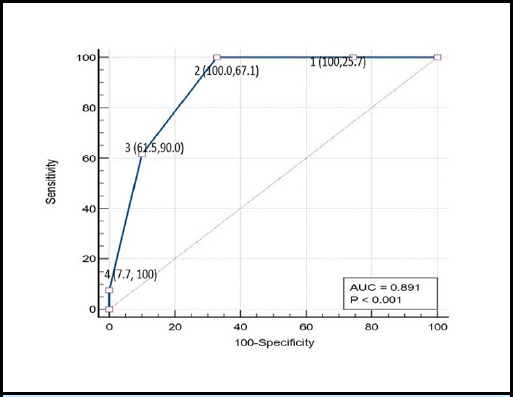
ROC curve of DECAF score for predicting inhospital mortality (n=83).

**Figure 3:**
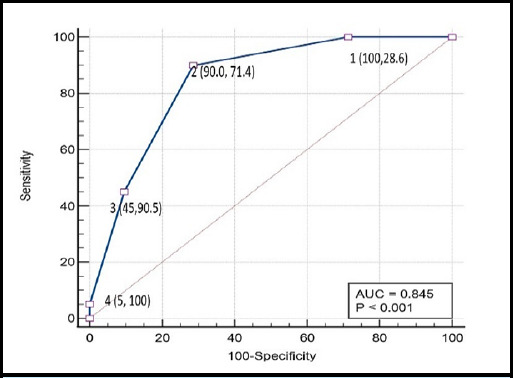
ROC curve of DECAF score for predicting need of ICU stay (n=83).

The AUROC of DECAF score for inhospital mortality was 0.891 (p < 0.001) and 0.845 (p < 0.001) for need of ICU stay (Figure 2 and 3).

## DISCUSSION

A total of 83 patients were included in our study as per our patient selection methods, inclusion and exclusion criteria. The mean age of the patients was 69.1 years. Out of 83 patients, 57 (68.67%) were female showing more female prevalence among hospitalized patients. It is possible that this is attributable to increased exposure to household smoke resulting from the use of firewood. Most studies have reported that COPD prevalence and mortality are greater among men than women, but later data from developed countries has reported that the prevalence of COPD is now almost equal in men and women, probably reflecting the changing patterns of tobacco smoking. ^11^ The data from GBD (Global Burden of Disease) repository presenting morbidity and mortality attributed to COPD by sex and age in Nepal also showed higher prevalence among males but lower DALYs (Disability-adjusted life years).^12^ Although controversial, some studies have even suggested that women are more susceptible to the effects of tobacco smoke than men,^13,14^ leading to more severe disease for the equivalent quantity of cigarettes consumed.

Our study showed a mortality rate of 15.66%, a result that lies within the range of 6-20% that has been reported in studies done in Nepal.^4,5^ The inhospital death rate shows a wide range within different countries with mortality of one out of 12 in a study done in UK in 2008 and one out of 40 in U.S in 1996. In the study done in ZAGAZIG university, Egypt,^15^ the mortality rate was 12.5% whereas different studies have showed the range of 4-30% of inhospital mortality.^16^ This might indicate a difference in the threshold for hospital admission and care between the countries. Of all the patients, 20 (24%) required ICU care, with 7 deaths among them which might have influenced the mortality rate of our study. These results emphasize the importance of timely access to specialized medical treatment and highlight the difficulties faced by healthcare providers in managing complex cases. The varying range of mortality rates depicts a significant heterogeneity in the clinical presentation, risk factors, exposures, and clinical outcomes in patients with AECOPD.^17^ It's probable that differences in patient characteristics and variations in the quality of care can explain much of the observed variability.

Among the 13 patients who died, majority had a DECAF score of 2 or 3. There was no mortality in patients with DECAF of 0 and 1 and there were no patients with DECAF score of 5 or 6. In various studies^7,9,15^ there were around 2% of all patient with DECAF of 5 whereas only few patients had DECAF of 6. The sample size in this study may have been small to observe patients with higher DECAF scores. The patients in our study may have different demographic or clinical characteristics compared to other populations where patients with higher DECAF scores are more commonly observed. It is also possible that, in some cases, patients with more severe COPD may not seek medical attention until it's too late, may lack family/social support or they may not have access to healthcare facilities due to factors such as distance, transportation, or cost.

In terms of AUC our study showed that DECAF score is statistically significant for predicting outcomes of inpatient mortality and need of ICU stay. In present study, DECAF score showed an excellent discrimination for in hospital mortality AUROC = 0.891, 95% CI 0.803 to 0.949 which is comparable to previous studies.^7,9,15^ The DECAF showed similar result for need of ICU stay with AUROC = 0.845, 95% CI 0.749 to 0.915. The DECAF score cutoff of two or more would successfully detect 100% of mortality cases, with a specificity of 67.1%. This result is similar to a 2017 Indian study on 150 AECOPD patients.^18^ Similarly, DECAF score of two would also be an ideal cutoff for predicting need of ICU stay with sensitivity of 90% and specificity of 71.4%.

Considering the mortality rates for each DECAF score, hospitalized AECOPD patients can be stratified into three risk categories: DECAF 0 - 1(low risk; mortality = 0%); DECAF 2 (moderate risk; inhospital mortality = 23.80%); DECAF 3 - 6 (high risk; mortality 53.33%). Findings of this study suggests that nearly half (56.62%) of the patients with AECOPD can be classified as low risk (DECAF 0-1) of inhospital mortality and therefore potentially be suitable for early supported discharge. On the other hand, a high DECAF score (≥2) might be used as a guide to early escalation of care.

In our study, only the DECAF score was analyzed. It would have been more informative if DECAF score was compared with scores such as CURB65, BAP65, APACHE II and CAPS. In this study, clinical diagnosis of COPD was considered rather than spirometry which is the standard requirement. As all patients being treated as COPD did not have initial spirometry diagnosis and also considering irregularity of spirometry due to covid pandemic state, only clinical diagnosis was considered as per GOLD criteria. The sample size in the study might have been small to observe patients with higher DECAF scores and patients with eMRCD scale of 5b. Convenience sampling in this study may not accurately represent all patients with AECOPD. The sample could over-represent certain patients such as those with more severe disease, thus inflating the sensitivity observed. A more representative sampling method, like random sampling, would help ensure the findings are generalizable to all AECOPD patients.

## CONCLUSIONS

This study shows that DECAF score is a good predictor of inhospital mortality and ICU admission.
